# Dermatofibrosarcoma Protuberans (DFSP) with Fibrosarcomatous Changes in a Patient with Crohn's Disease Treated with Anti-TNF (Adalimumab)

**DOI:** 10.1155/2023/1057247

**Published:** 2023-10-10

**Authors:** Ivo Klarin

**Affiliations:** ^1^General Hospital Zadar, Department of Gastroenterology, Zadar, Croatia; ^2^University of Zadar, Department for Health Studies, Zadar, Croatia

## Abstract

Dermatofibrosarcoma protuberans (DFSP) is a low-to-intermediate grade sarcoma with a reported incidence of 4.1 per million person-years. Despite its local invasiveness, it rarely metastasizes (5% of cases). Fibrosarcomatous change in DFSP is a form of tumor progression that carries an increased risk of metastases. We reported a case of 45-year-old patient treated with adalimumab lasting 7 years for Crohn's disease who developed dermatofibrosarcoma protuberans with fibromatous changes. Adalimumab therapy was stopped before surgery, and ustekinumab was introduced 6 months after.

## 1. Introduction

Crohn's disease (CD) is an inflammatory bowel disease affecting any portion of the gastrointestinal tract, usually the terminal ileum and the colon. Clinical manifestations of CD include diarrhea, fever, and weight loss, but it can also manifest with a wide range of extra-intestinal symptoms. Treatment modalities include steroids, antibiotics, immunosuppressants, biologics, and surgery. Biological therapy with anti-TNF-*α* agents offers significant therapeutic benefits, but their use requires caution, as they can also be associated with numerous side effects [1]. Here we describe a patient with Crohn's disease treated with adalimumab who developed dermatofibrosarcoma protuberans (DFSP).

## 2. Case Report

**Figure 1:**
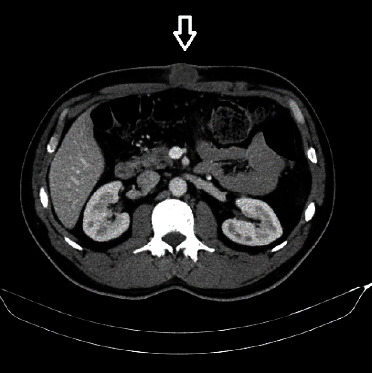
Cross-sectional computed tomography shows tumor in the abdominal wall.

**Figure 2:**
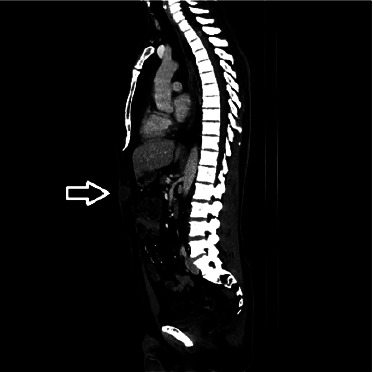
Sagittal computed tomography—tumor in the abdominal wall.

**Figure 3:**
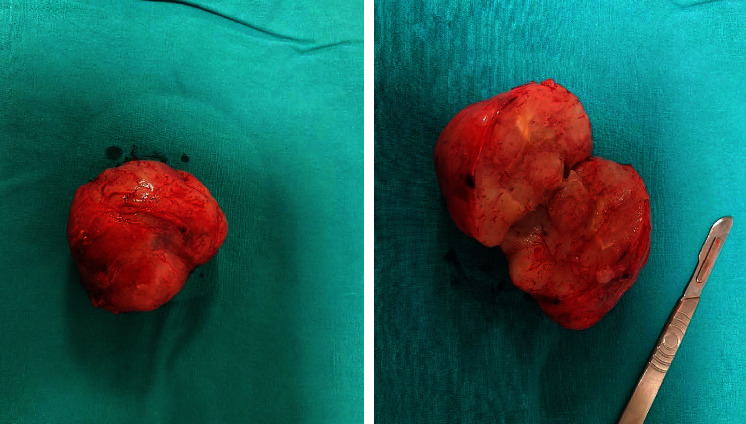
Dermatofibrosarcoma protuberans.

A 45-year-old Caucasian man with a history of Crohn's disease was treated with adalimumab injections, subcutaneous 40 mg every 2 weeks for the past 7 years. He was diagnosed with Crohn's disease of the terminal ileum 20 years ago. He was treated with azathioprine for 13 years, and he was in remission. During 2013, there was a clinical deterioration (pain and passage disturbances). An inflammatory stenosis of the terminal ileum (long segment) and transversum (short segment) was confirmed by MSCT and MR enterography. He was treated with systemic corticosteroids, which achieved significant clinical improvement. Biological therapy with adalimumab was started as maintenance therapy. For the next 3 years, the patient was in clinical remission, with no laboratory or endoscopic signs of inflammation. In 2016, pains due to obstruction of the passage appeared again, but without laboratory signs of inflammation. Repeated MR enterography confirmed previously known stenoses, which are now predominantly fibrous. A surgical resection of the terminal ileum was performed along with strictureplasty of the large intestine. Postoperatively, adalimumab therapy was continued; subjectively, he felt well (clinical, biochemical, and endoscopic remission). During therapy with adalimumab, the patient did not take any other oral medications. In 2020, the patient noticed a small painless growth at the site of the surgical incision (scar). Diagnostic evaluation was performed (transabdominal ultrasound and MSCT), and the formation was well limited, 5 × 3 cm in size (Figures 1 and 2). Due to the growth tendency, surgical excision was performed in September 2020 (Figure 3). Pathological analysis shows that it is a fibrosarcoma transformation of dermatofibrosarcoma protuberans (7.5 × 7 × 6 cm). Due to the presence of tumor at the edges, an additional wide surgical excision was performed. Histologically, the tumor is made up of hypercellular, long bundles of atypical mesenchymal cells with spindle-shaped nuclei and sparse cytoplasm (the so-called “herringbone pattern”). Tumor cells show strong nuclear atypia and high mitotic activity (up to 23 mitoses/10 WP). There are areas of coagulation necrosis (up to 20% of the surface). No vascular invasion is seen. Immunohistochemically, tumor cells give a positive reaction to CD34 and a negative reaction to S100, SMA, desmin, EMA, AE1/AE3, and CD117. It is a high-grade sarcoma (3 according to FNCLCC). The multidisciplinary team decided to discontinue adalimumab therapy, although there are no clear guidelines on anti-TNF drug therapy during the treatment of malignant disease. The patient was treated with adjuvant radiotherapy. Clinical worsening of Crohn's disease occurs 6 months after discontinuation of adalimumab therapy. It was decided to start ustekinumab therapy, and clinical and endoscopic remission was again achieved. Following up to now, 3 years after the surgical treatment, there is no recurrence of the malignant disease.

## 3. Discussion

DFSP is a low-to-intermediate grade sarcoma with a reported incidence of 4.1 per million person-years. Age at diagnosis is usually between 20 and 59 years, although it may be seen at any age, even congenitally. It is usually distributed on the trunk, more than the extremities or head and neck. Most patients had primary tumors characterized by a red or brownish red, protruding, well-circumscribed, and firm nodule [2]. It arises from the dermis and invades deeper subcutaneous tissues, but, despite its local invasiveness, it rarely metastasizes (5% of cases). Fibrosarcomatous change in DFSP is a form of tumor progression that carries an increased risk of metastases [3]. The origin of DFSP is unknown [4]. The treatment of DFSP is generally surgical, and wide local excision is the mainstay of surgical treatment [5, 6]. It is not clear whether in our case the development of this tumor was triggered or caused by TNF-alfa blocker therapy or represents an independent event. We can also speculate that the fibrosarcomatous changes may be related to adalimumab therapy. To our knowledge, despite the intense interest in evaluating the risk of cancer associated with TNF inhibitors, there were only two reported cases of DFSP or DSFP with fibrosarcomatous features in patients on TNF-alfa blocker treatment, but there were no patients with Crohn's disease [7, 8]. Given the rarity of DFSP and its prior association with immune suppression, a case in a patient treated with a TNF inhibitor may suggest a safety signal. Additional reports will be necessary for further investigation. Patients receiving chronic TNF inhibitors should be carefully monitored for skin malignancies. The question of the choice of Crohn's disease therapy for these patients (anti-TNF, ustekinumab, and vedolizumab) is still unanswered [9, 10].

## Data Availability

All data generated or analyzed during this study are included in this article. Further enquiries can be directed to the corresponding author.
